# Supplementary material for: Vagus-macrophage-hepatocyte link promotes post-injury liver regeneration and whole-body survival through hepatic FoxM1 activation
Source: Nat Commun. 2018 Dec 13;9:5300. doi: 10.1038/s41467-018-07747-0 (PMC6294142; doi:10.1038/s41467-018-07747-0)
Supplement: Supplementary file 1 — Supplementary Information [file 41467_2018_7747_MOESM1_ESM.pdf]

Supplementary Information
Supplementary Figures 1-6
Supplementary Table 1

Vagus-macrophage-hepatocyte link promotes post-injury liver regeneration and whole-body survival through hepatic FoxM1 activation

Izumi et al.

Izumi et al. Supplementary Figure 1

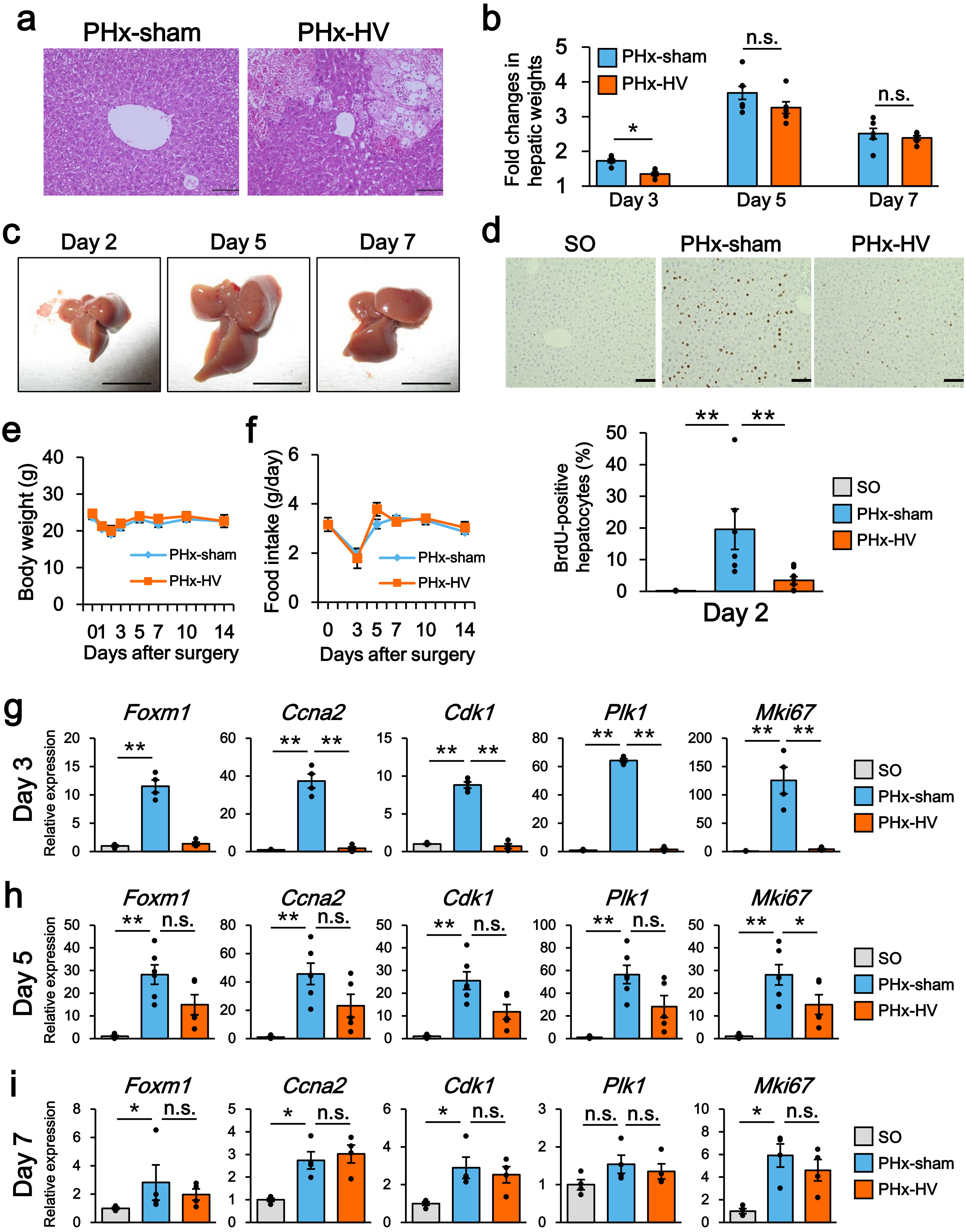

Supplementary Figure 1

Hepatic branch vagotomy suppresses liver regeneration at the early stage after partial hepatectomy

(a) Representative images of liver sections stained with hematoxylin and eosin from survived PHx-sham-mice and PHx-HV-mice which died 2 days after the operation. Scale bars indicate 100 μm.

(b) Fold changes in hepatic weights 3, 5 and 7 days after surgery in PHx-sham-mice (n = 5 - 6 per group) and PHx-HV-mice (n = 5 per group). Hepatic weights at both indicated time points were divided by those obtained immediately after surgery.

(c) Representative macro images of the liver extracted from mice 2, 5 and 7 days after PHx-sham. Scale bars indicate 10 mm.

(d) (Left panels) Representative images of liver sections immunostained for BrdU on day 2 after sham operation for PHx (SO) and PHx. Samples were collected from mice that underwent SO and PHx 10 days after HV or sham operation for HV. Scale bars indicate 100 μm. (Right panel) BrdU-positive hepatocyte ratios in SO-mice (n = 6), PHx-sham-mice (n = 6) and PHx-HV-mice (n = 8) on postoperative day 2.

(e and f) Body weights (e) and food intakes (f) after surgery in PHx-sham-mice (n = 4) and PHx-HV-mice (n = 5).

(g) Relative expressions of *Foxm1*, its target genes and *MKi67* in the liver 3 days after sham operation for PHx (SO) (n = 4), PHx-sham (n = 4) and PHx-HV (n = 4).

(h) Relative expressions of *Foxm1*, its target genes and *MKi67* in the liver 5 days after SO (n = 5), PHx-sham (n = 6) and PHx-HV (n = 5).

(i) Relative expressions of *Foxm1*, its target genes and *MKi67* in the liver 7 days after SO (n = 4), PHx-sham (n = 4) and PHx-HV (n = 4).

Data are presented as means ± SEM.

*$P < 0.05$; **$P < 0.01$ assessed by unpaired *t*-test (b, e, f) or assessed by one-way ANOVA followed by Bonferroni's post hoc test (d, g, h, i). n.s., not significant.

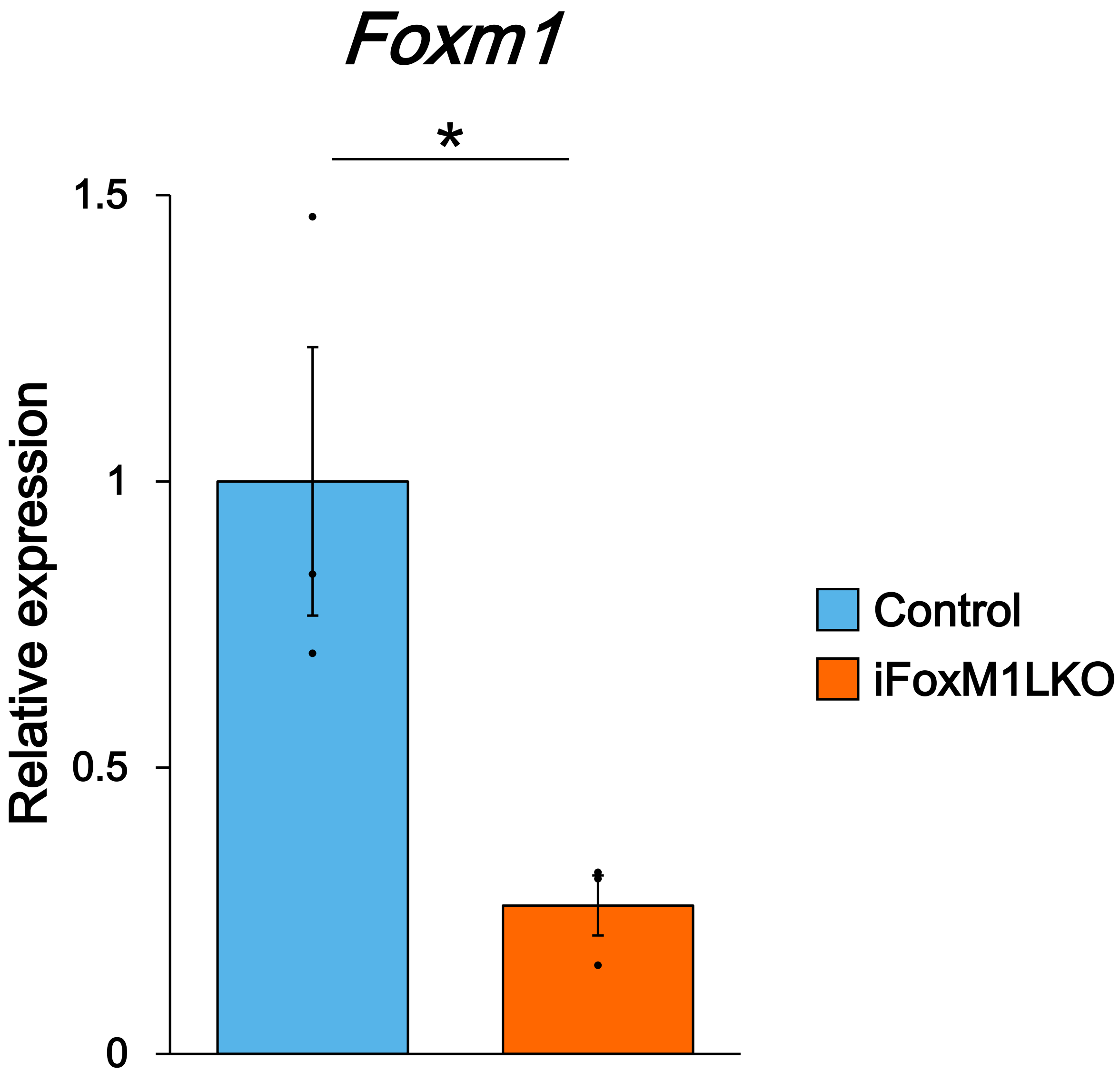
Foxm1
*
1.5
1
0.5
0
Relative expression
Control
iFoxM1LKO

Supplementary Figure 2

Efficacy of FoxM1 knockout by tamoxifen-induced Cre-mediated recombination in the liver

Expressions of the *Foxm1* gene in the livers of control (n = 3) and iFoxM1LKO mice (n = 3) on day 7 after the end of tamoxifen injection.

Data are presented as means ± SEM.

$*P < 0.05$ assessed by unpaired *t*-test.

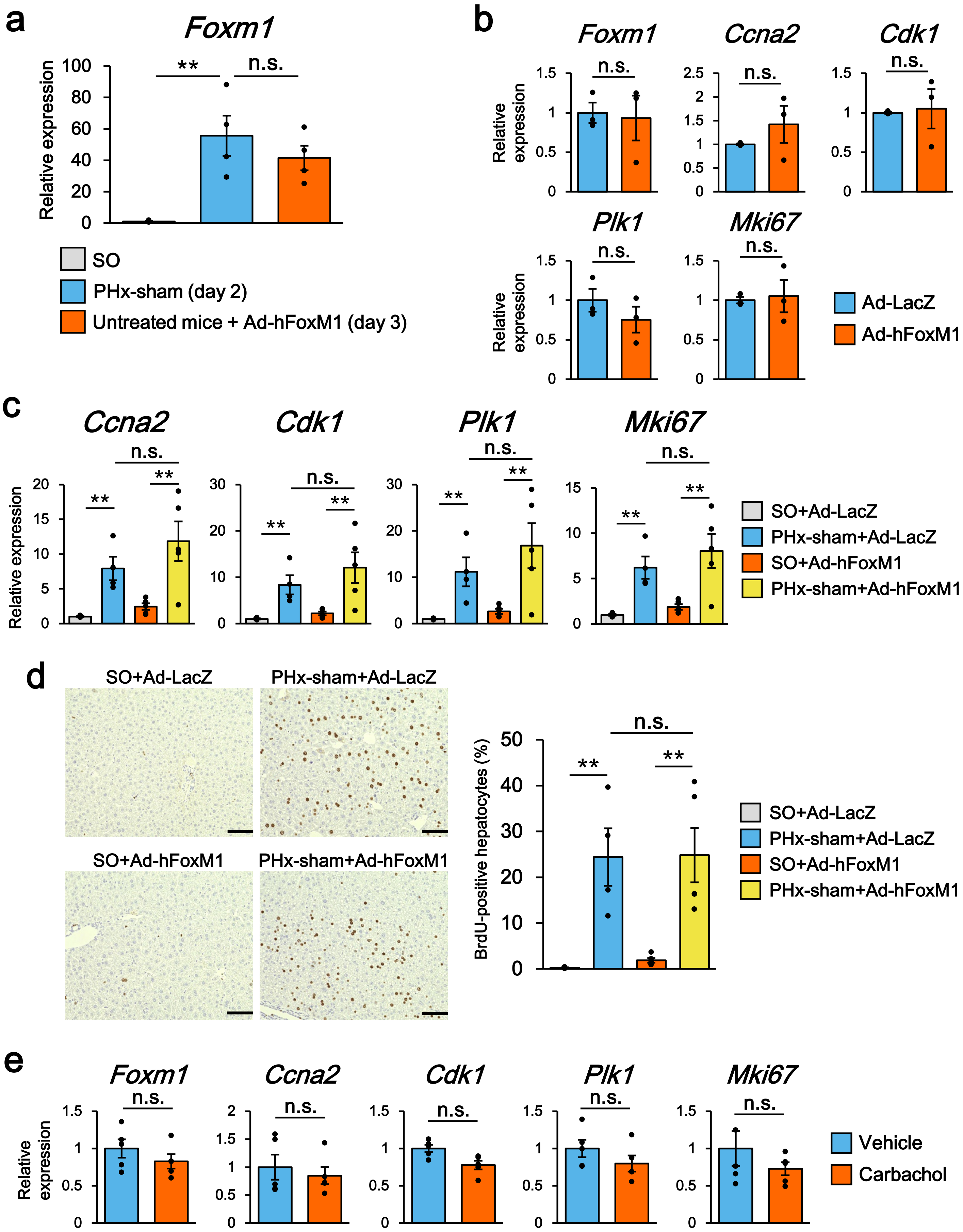
a
Foxm1
Relative expression
100
80
60
40
20
0
**
n.s.
SO
PHx-sham (day 2)
Untreated mice + Ad-hFoxM1 (day 3)
b
Foxm1
Ccna2
Cdk1
Plk1
Mki67
Relative expression
n.s.
Ad-LacZ
Ad-hFoxM1
c
Ccna2
Cdk1
Plk1
Mki67
Relative expression
SO+Ad-LacZ
PHx-sham+Ad-LacZ
SO+Ad-hFoxM1
PHx-sham+Ad-hFoxM1
d
SO+Ad-LacZ
PHx-sham+Ad-LacZ
SO+Ad-hFoxM1
PHx-sham+Ad-hFoxM1
BrdU-positive hepatocytes (%)
50
40
30
20
10
0
e
Foxm1
Ccna2
Cdk1
Plk1
Mki67
Relative expression
Vehicle
Carbachol

Supplementary Figure 3

Endogenous FoxM1 pathway is not affected by adenoviral FoxM1 supplementation or carbachol administration in normal mice

(a) Expressions of murine *Foxm1* gene in the liver 2 days after sham operation for PHx (SO) (n = 4) and PHx-sham (n = 4), and human *Foxm1* gene in the liver 3 days after adenoviral gene transduction (n = 4).

(b) Relative expressions of *Foxm1*, its target genes and *MKi67* in the liver from mice 3 days after receiving Ad-LacZ (n = 3) or Ad-hFoxM1 (n = 3) without any surgical interventions.

(c) Relative expressions of *Foxm1*, its target genes and *MKi67* in the liver 2 days after SO and PHx-sham in mice receiving Ad-LacZ (n = 4 -5 per group) and Ad-hFoxM1 (n = 4 - 5 per group)

(d) (Left panels) Representative images of liver sections immunostained for BrdU on day 2 after SO and PHx-sham in mice receiving Ad-LacZ and Ad-hFoxM1. Scale bars indicate 100 µm. (Right panel) BrdU-positive hepatocyte ratios in the liver 2 days after SO and PHx-sham in mice receiving Ad-LacZ (n = 4 -5 per group) and Ad-hFoxM1 (n = 4 - 5 per group).

(e) Relative expressions of *Foxm1*, its target genes and *MKi67* in the liver from mice 6 hours after administration of vehicle (n = 5) or carbachol (n = 5) without any surgical interventions.

Data are presented as means ± SEM.

**$P < 0.01$ assessed by one-way ANOVA followed by Bonferroni's post hoc test (a, c, d) or assessed by unpaired *t*-test (b, e). n.s., not significant.

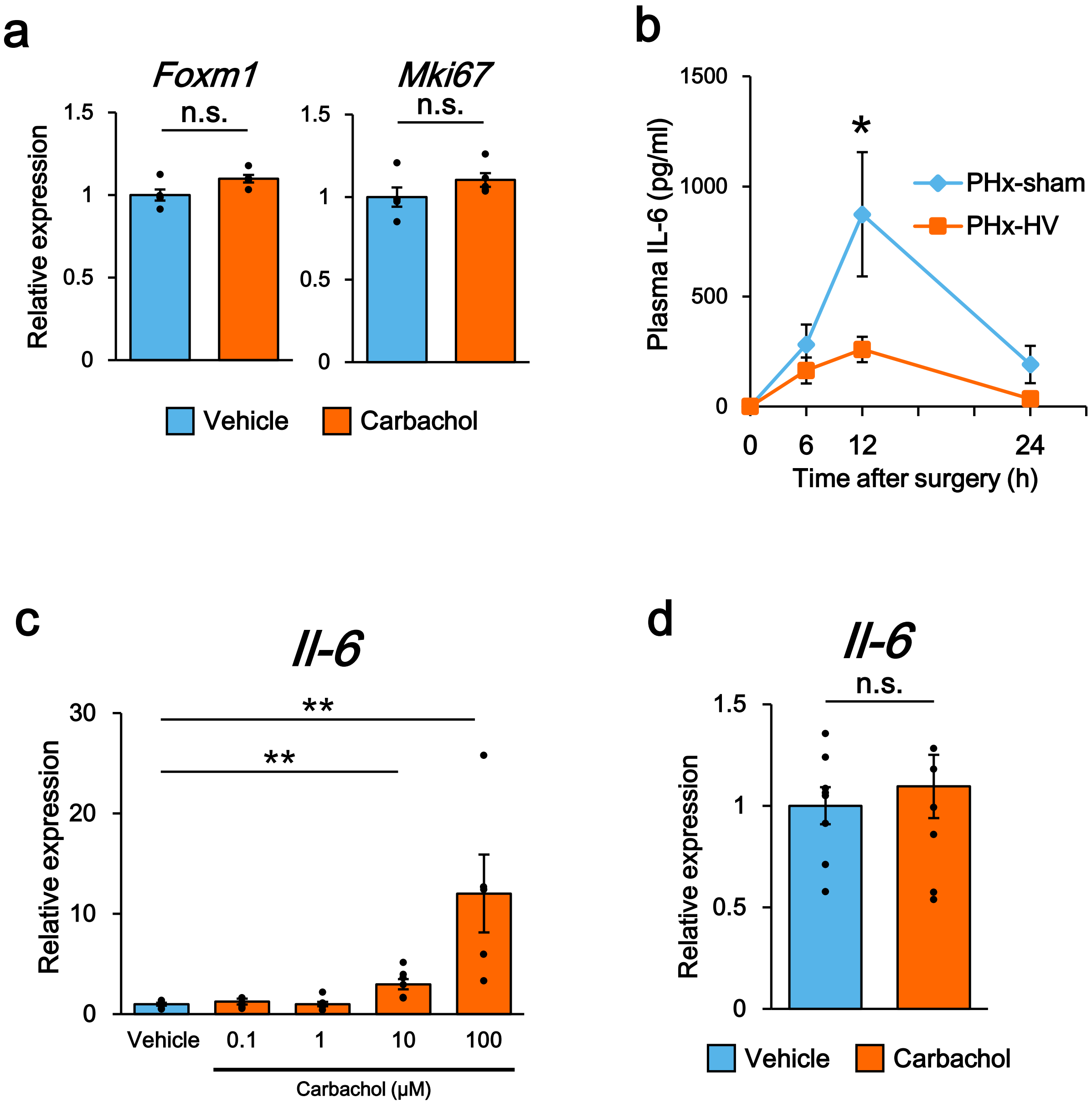
a
Foxm1
Mki67
n.s.
n.s.
Relative expression
1.5
1
0.5
0
Vehicle
Carbachol
b
Plasma IL-6 (pg/ml)
1500
1000
500
0
*
PHx-sham
PHx-HV
0
6
12
24
Time after surgery (h)
c
Il-6
Relative expression
30
20
10
0
**
**
Vehicle
0.1
1
10
100
Carbachol (μM)
d
Il-6
n.s.
Relative expression
1.5
1
0.5
0
Vehicle
Carbachol

Supplementary Figure 4

Vagal signals or carbachol treatment upregulate IL-6 expression in macrophages, but not in hepatocytes

(a) Expressions of *Foxm1* and *Mki67* genes in Hepa1-6 cells treated with 100 µM carbachol (n = 5) or vehicle (n = 4) for 24 hours.

(b) Plasma IL-6 concentrations of PHx-sham- (n = 5) and PHx-HV-mice (n = 6) at 0, 6, 12 and 24 hours after the operation.

(c) Relative gene expressions of *Il-6* in primary macrophages after stimulation with vehicle (n = 6) or 0.1, 1, 10 and 100 µM concentrations of carbachol (n = 5 - 7 per group) for 4 hours.

(d) Relative gene expressions of *Il-6* in primary hepatocytes after stimulation with vehicle (n = 8) or 100 µM carbachol (n = 8) for 4 hours.

Data are presented as means ± SEM.

*$P < 0.05$; **$P < 0.01$ assessed by unpaired *t*-test (a, d, f) or assessed by one-way ANOVA followed by Bonferroni's post hoc test (e). n.s., not significant.

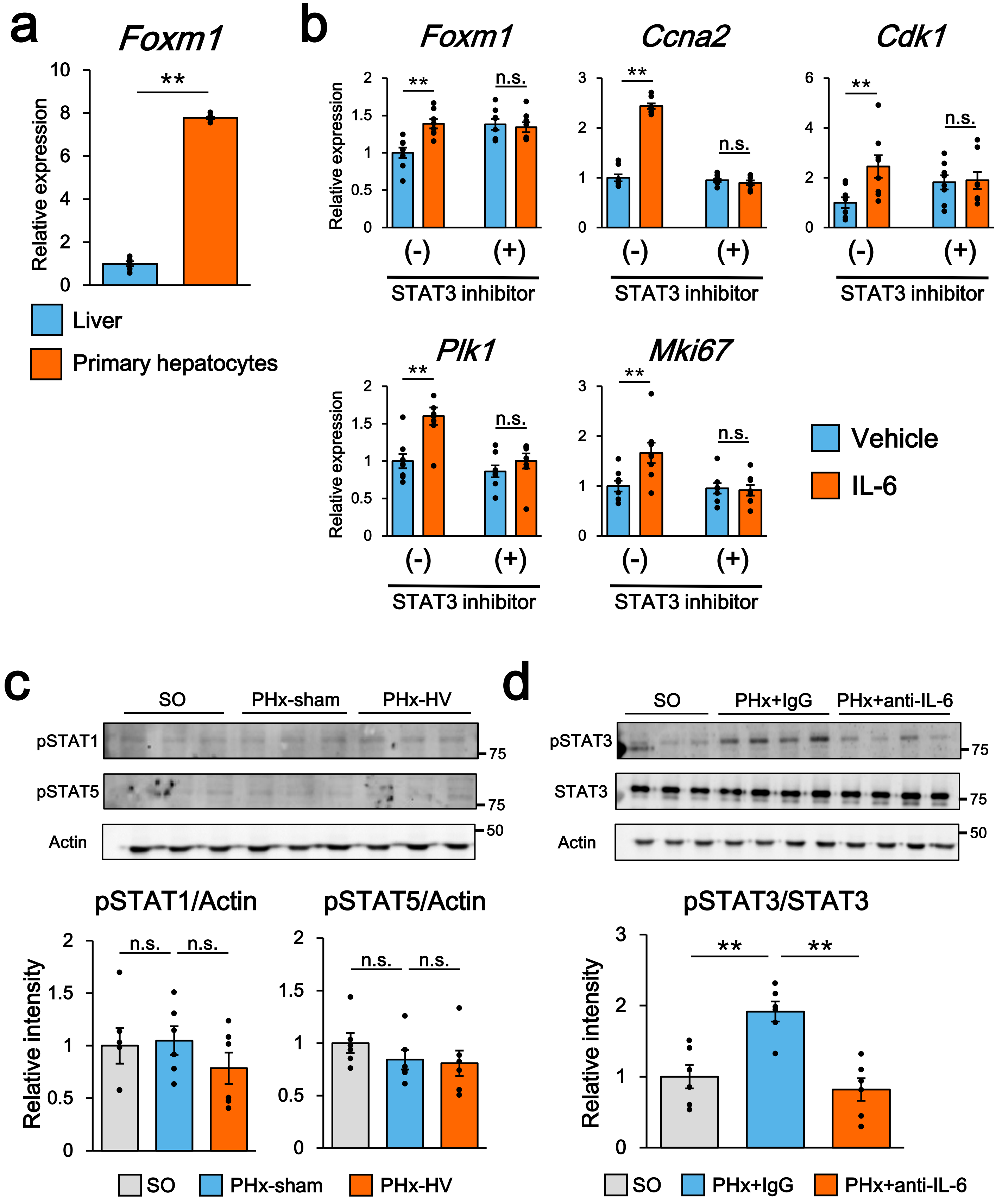
a
Foxm1
Relative expression
**
Liver
Primary hepatocytes
b
Foxm1
Ccna2
Cdk1
Plk1
Mki67
Relative expression
**
n.s.
(-)
(+)
STAT3 inhibitor
Vehicle
IL-6
c
SO
PHx-sham
PHx-HV
pSTAT1
pSTAT5
Actin
75
50
pSTAT1/Actin
pSTAT5/Actin
Relative intensity
n.s.
d
SO
PHx+IgG
PHx+anti-IL-6
pSTAT3
STAT3
Actin
75
50
pSTAT3/STAT3
Relative intensity
**

Supplementary Figure 5

IL-6-mediated STAT3 activation is required for liver regeneration after PHx

(a) Expressions of the *Foxm1* gene in non-treated whole liver (n = 6) and primary hepatocytes 48 hours after isolation (n = 8).

(b) Relative expressions of *Foxm1*, its target genes and *MKi67* in primary hepatocytes stimulated with 100 ng/ml IL-6 (n = 8 per group) or vehicle (n = 8 per group) for 6 hours with or without pretreatment with STAT3 inhibitor peptide.

(c) (Upper panels) Representative images of liver extract immunoblottings with anti-phospho-STAT1, phospho-STAT5 and actin. (Lower panels) Relative intensities of phospho-STAT1/actin and phopsho-STAT5/actin in the livers from sham operation for PHx (SO)- (n = 6), PHx-sham- (n = 6) and PHx-HV-mice (n = 6).

(d) (Upper panels) Representative images of liver extract immunoblottings with anti-phospho-STAT3, total STAT3 and actin. (Lower panel) Relative intensities of phospho/total STAT3 in livers from sham operation for PHx (SO)- (n = 6), PHx-sham- (n = 6) and PHx-HV-mice (n = 6).

Data are presented as means ± SEM.

***P* < 0.01 assessed by unpaired *t*-test (a, b), or one-way ANOVA followed by Bonferroni's post hoc test (c, d).

Izumi et al. Supplementary Figure 6

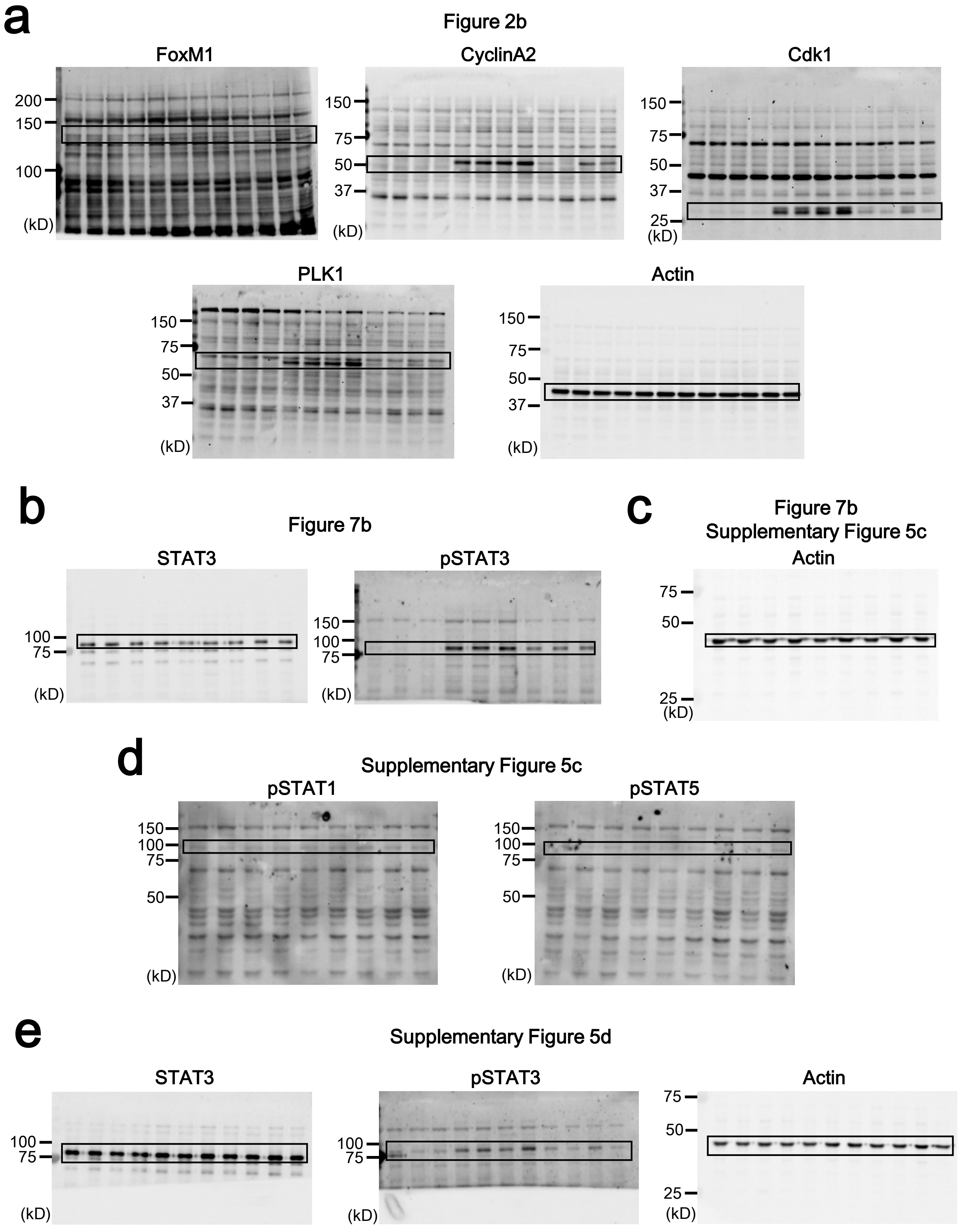

Supplementary Figure 6

Uncropped scans of the immunoblots for Figures 2b and 7b, Supplementary Figures 5c and 5d

	forward (5' to 3')	reverse (5' to 3')
Mouse Foxm1	GCTCCATAGAAATGTGACCATC	AACCTTCACTGAGGGCTGTAAC
Mouse Cdk1	AAGAACCTGGACGAGAACG	GTCATCAAAGTACGGGTGCT
Mouse Ccna2	CCTTAAGTACCTGCCTTCACTC	ACAAGGCTTAAGACTCTCCA
Mouse Plk1	ACGGCACCGTGCAGATTA	AGGCGGTACGTTTGGAAGTC
Mouse Mki67	AGTCTCTGGAGAGTCTGATGTTA	ACTTCTTGGTGCATACAATGTC
Mouse F4/80	CTTTGGCTATGGGCTTCCAGTC	GCAAGGAGGACAGAGTTTATCGTG
Mouse Il6	CGTGGAAATGAGAAAAGAGTTG	ATCCAGTTTGGTAGCATCCATC
Mouse Actb	GATGCCCTGAGGCTCTT	TGTGTTGGCATAGAGGTCTTTAC
Human FoxM1	GCAGAACAGCGTGTCCGAGA	CGGGTTTGGCGATATGCTTG

Supplementary Table 1

Sequences of RT-PCR oligonucleotide primers
