# Supplementary material for: Vagus-macrophage-hepatocyte link promotes post-injury liver regeneration and whole-body survival through hepatic FoxM1 activation
Source: Nat Commun. 2018 Dec 13;9:5300. doi: 10.1038/s41467-018-07747-0 (PMC6294142; doi:10.1038/s41467-018-07747-0)
Supplement: Supplementary file 3 — Reporting Summary [file 41467_2018_7747_MOESM3_ESM.pdf]

nature research

Corresponding author(s): Junta Imai

Reporting Summary

Nature Research wishes to improve the reproducibility of the work that we publish. This form provides structure for consistency and transparency in reporting. For further information on Nature Research policies, see Authors & Referees and the Editorial Policy Checklist.

Statistical parameters

When statistical analyses are reported, confirm that the following items are present in the relevant location (e.g. figure legend, table legend, main text, or Methods section).

n/a	Confirmed	
☐	☒	The exact sample size (*n*) for each experimental group/condition, given as a discrete number and unit of measurement
☐	☒	An indication of whether measurements were taken from distinct samples or whether the same sample was measured repeatedly
☐	☒	The statistical test(s) used AND whether they are one- or two-sided<br>*Only common tests should be described solely by name; describe more complex techniques in the Methods section.*
☐	☒	A description of all covariates tested
☐	☒	A description of any assumptions or corrections, such as tests of normality and adjustment for multiple comparisons
☐	☒	A full description of the statistics including central tendency (e.g. means) or other basic estimates (e.g. regression coefficient) AND variation (e.g. standard deviation) or associated estimates of uncertainty (e.g. confidence intervals)
☒	☐	For null hypothesis testing, the test statistic (e.g. *F*, *t*, *r*) with confidence intervals, effect sizes, degrees of freedom and *P* value noted<br>*Give P values as exact values whenever suitable.*
☒	☐	For Bayesian analysis, information on the choice of priors and Markov chain Monte Carlo settings
☒	☐	For hierarchical and complex designs, identification of the appropriate level for tests and full reporting of outcomes
☒	☐	Estimates of effect sizes (e.g. Cohen's *d*, Pearson's *r*), indicating how they were calculated
☐	☒	Clearly defined error bars<br>*State explicitly what error bars represent (e.g. SD, SE, CI)*

Our web collection on statistics for biologists may be useful.

Software and code

Policy information about availability of computer code

Data collection	*Provide a description of all commercial, open source and custom code used to collect the data in this study, specifying the version used OR state that no software was used.*
Data analysis	*Provide a description of all commercial, open source and custom code used to analyse the data in this study, specifying the version used OR state that no software was used.*

For manuscripts utilizing custom algorithms or software that are central to the research but not yet described in published literature, software must be made available to editors/reviewers upon request. We strongly encourage code deposition in a community repository (e.g. GitHub). See the Nature Research guidelines for submitting code & software for further information.

Data

Policy information about availability of data

All manuscripts must include a data availability statement. This statement should provide the following information, where applicable:

- Accession codes, unique identifiers, or web links for publicly available datasets
- A list of figures that have associated raw data
- A description of any restrictions on data availability

The data that support the findings of this study are available from the corresponding author upon reasonable request.

Field-specific reporting

Please select the best fit for your research. If you are not sure, read the appropriate sections before making your selection.

- [x] Life sciences
- [ ] Behavioural & social sciences
- [ ] Ecological, evolutionary & environmental sciences

For a reference copy of the document with all sections, see nature.com/authors/policies/ReportingSummary-flat.pdf

Life sciences study design

All studies must disclose on these points even when the disclosure is negative.

Sample size	Most sample sizes were chosen based on data from previous publications.
Data exclusions	No data were excluded from analyses.
Replication	All attempts at replication were successful.
Randomization	Statistical analyses were performed assuming a normal distribution in all experiments.
Blinding	The investigators were not blinded to group allocation during data collection.

Reporting for specific materials, systems and methods

Materials & experimental systems

n/a	Involved in the study	
☒	☐	Unique biological materials
☐	☒	Antibodies
☐	☒	Eukaryotic cell lines
☒	☐	Palaeontology
☐	☒	Animals and other organisms
☒	☐	Human research participants

Methods

n/a	Involved in the study	
☒	☐	ChIP-seq
☒	☐	Flow cytometry
☒	☐	MRI-based neuroimaging

Antibodies

Antibodies used	Antibodies to phospho-STAT3 (Tyr705) (#9131, Cell Signaling Technology, Danvers, MA, USA), total STAT3 (#4904, Cell Signaling Technology), phospho-STAT1 (Tyr701) (#9167, Cell Signaling Technology), phospho-STAT5 (Tyr694) (#9314, Cell Signaling Technology) , FoxM1 (13147-1-AP, Proteintech, Rosemont, IL, USA), Cyclin A2 (ab181591, Abcam, Cambridge, UK), Cdk1 (#28439, Cell Signaling Technology) and PLK1 (#4535, Cell Signaling Technology), actin (A2066, Sigma), BrdU (51-75512 L, BD Bioscience) and F4/80 antibody (12-4801-80, Affymetrix eBioscience, Santa Clara, CA, USA)
Validation	Validation statements of all antibodied are on the manufacture's websites.

Eukaryotic cell lines

Policy information about cell lines

Cell line source(s)	Hepa1-6 cells.
Authentication	Hepa1-6 cells were authenticated by ATCC.
Mycoplasma contamination	Hepa1-6 cells were not tested for mycoplasma contamination.
Commonly misidentified lines (See ICLAC register)	*Name any commonly misidentified cell lines used in the study and provide a rationale for their use.*

Animals and other organisms

Policy information about studies involving animals; ARRIVE guidelines recommended for reporting animal research

Laboratory animals	C57BL/6N mice: male, 8 weeks of age. SA-CreERT2; FoxM1flox/flox mice: male, 8 weeks of age
Wild animals	*Provide details on animals observed in or captured in the field; report species, sex and age where possible. Describe how animals were caught and transported and what happened to captive animals after the study (if killed, explain why and describe method; if released, say where and when) OR state that the study did not involve wild animals.*
Field-collected samples	*For laboratory work with field-collected samples, describe all relevant parameters such as housing, maintenance, temperature, photoperiod and end-of-experiment protocol OR state that the study did not involve samples collected from the field.*
